# Vascular Imaging Techniques to Diagnose and Monitor Patients with Takayasu Arteritis: A Review of the Literature

**DOI:** 10.3390/diagnostics11111993

**Published:** 2021-10-27

**Authors:** Kazumasa Oura, Mao Yamaguchi Oura, Ryo Itabashi, Tetsuya Maeda

**Affiliations:** Division of Neurology and Gerontology, Department of Internal Medicine, School of Medicine, Iwate Medical University, 2-1-1 Idaidori, Yahaba-Cho, Shiwa-Gun, Morioka 028-3695, Japan; maooura@iwate-med.ac.jp (M.Y.O.); ritabash@iwate-med.ac.jp (R.I.); maeda@iwate-med.ac.jp (T.M.)

**Keywords:** Takayasu arteritis, large vessel vasculitis, stroke, imaging techniques, monitoring

## Abstract

Takayasu arteritis (TA) is a large vessel vasculitis that causes stenosis, occlusion, and sometimes the aneurysm of the aorta and its major branches. TA often occurs in young women, and because the symptoms are not obvious in the early stages of the disease, diagnosis is difficult and often delayed. In approximately 10% to 20% of patients, TA is reportedly complicated by ischemic stroke or transient ischemic attack. It is important to diagnose TA early and provide appropriate treatment to prevent complications from stroke. Diagnostic imaging techniques to visualize arterial stenosis are widely used in clinical practice. Even if no signs of cerebrovascular events are present at the time of the most recent evaluation of patients with TA, follow-up vascular imaging is important to monitor disease progression and changes in the cerebrovascular risk. However, the optimal imaging technique for monitoring of TA has not been established. Therefore, the purpose of this review is to describe newly available evidence on the usefulness of conventional imaging modalities (digital subtraction angiography, computed tomography angiography, magnetic resonance imaging/angiography, duplex ultrasound, and positron emission tomography) and novel imaging modalities (optical coherence tomography, infrared thermography, contrast-enhanced ultrasonography, and superb microvascular imaging) in the diagnosis and monitoring of TA.

## 1. Introduction

Takayasu arteritis (TA) was first reported as a case of retinal vasculitis with pulselessness in 1908 by Mikito Takayasu, a Japanese ophthalmologist [1]. TA is a chronic large vessel vasculitis characterized by stenosis, occlusion, and sometimes the aneurysm of the aorta and its main branches, especially the subclavian artery, common carotid artery (CCA), and internal carotid artery [2]. TA often occurs in young women under the age of 40 years [3]. Early in the course of the disease, symptoms can be nonspecific, making diagnosis difficult and often delayed [4]. In approximately 10% to 20% of patients, TA is reportedly complicated by ischemic stroke or transient ischemic attack (TIA) [5]. It is important to diagnose TA early and provide appropriate treatment to prevent complications from stroke.

### 1.1. Pathophysiology of TA

TA is a systemic inflammatory disease that affects the aorta, its major branches, and major arteries, including the pulmonary artery [6,7]. Inflammatory lesions in patients with TA lead to thickening of the arterial wall and remodeling of the arterial lumen. Most often, arterial wall thickening and stenosis/occlusion occur; however, aneurysmal dilatation and arterial dissection are also possible [8,9,10,11]. TA is histologically characterized as “pan-arteritis,” in which all layers of the arterial wall are affected. Arteritis in TA causes neovascularization, leukocyte infiltration with arterial wall edema, degeneration of smooth muscle and elastic components, fibrosis, and hyperplasia of fibroblasts and myofibroblasts [8]. Macroscopically, this is accompanied by wall thickening, causing arterial stenosis or dilation, which directly affects clinical features and prognosis [8]. Specifically, TA includes the fibrous thickening of the intima and/or typical atheromatous lesions, destruction of smooth muscle and elastic layers along with cellular infiltration and fibrosis of the media, as well as thickening of the adventitia with cellular infiltration around vasa vasorum [12]. The rapid destruction of smooth muscle cells and elastic fibers in the media may lead to the formation of an aneurysm and/or dissecting aneurysm [13]. Abnormalities in the carotid artery or subclavian artery may cause cerebrovascular events such as ischemic stroke or TIA; therefore, particular attention to these arteries is required in patients with TA [14,15,16].

### 1.2. TA and Stroke Risk

Stroke remains the second leading cause of death and disability worldwide [17]. Patients with TA have an increased risk of stroke, and a meta-analysis of 21 studies (*n* = 3269 patients) reported that the pooled prevalence rate of stroke/TIA in patients with TA was 15.8% (95% confidence interval [CI]: 10.7–22.6%) [18]. With stroke becoming a major burden for patients, their families, and society worldwide [17], it is important to prevent/predict stroke in patients with TA.

### 1.3. Importance of Follow-Up Vascular Imaging in TA

Diagnostic imaging techniques to visualize arterial stenosis are widely used in clinical practice. Importantly, however, most patients with TA are diagnosed in emergency settings because ischemic stroke is sometimes the first clinical manifestation of TA [19,20,21]. Therefore, it is important to suspect TA in cases of juvenile stroke.

Even when no signs of cerebrovascular events are present at the time of the most recent evaluation of patients with TA, follow-up vascular imaging is important to monitor disease progression and identify changes in the risk of cerebrovascular events. A recently published systematic literature review of 287 articles showed no evidence of the optimal disease monitoring scheme [22]. Assessment of disease activity and damage in TA is problematic given the chronic, indolent disease course and lack of specific laboratory, and imaging findings [23]. Recommendations to guide monitoring and treatment of patients with TA are mainly obtained from observational studies with low levels of evidence [22]. Therefore, higher-quality studies are needed in the future.

Nevertheless, we recently reported a case of TA in a patient who presented with a thrombosed aneurysm of the right common carotid artery and developed cerebral infarction after neck massage [24]. In that case, if the patient had undergone follow-up imaging studies, the risk of embolism could have been predicted. Thus, we suggest that careful follow-up with carotid imaging should be considered for patients with TA to ensure timely detection of aneurysmal dilation and intra-aneurysmal thrombi of the carotid artery [24].

The purpose of this review is to describe the newly available evidence on the usefulness of conventional and novel imaging modalities for diagnosis and monitoring of TA.

## 2. Conventional Imaging Techniques for Diagnosis and Monitoring of TA

### 2.1. Digital Subtraction Angiography (DSA)

Conventional angiography was historically considered the best method for diagnosing TA [25]. With the development of less invasive imaging techniques, the role of conventional DSA is changing from a diagnostic tool to a therapeutic option with the implementation of endovascular treatment [26]. 

### 2.2. Computed Tomography Angiography (CTA)

#### 2.2.1. Diagnosis

**Figure 1:**
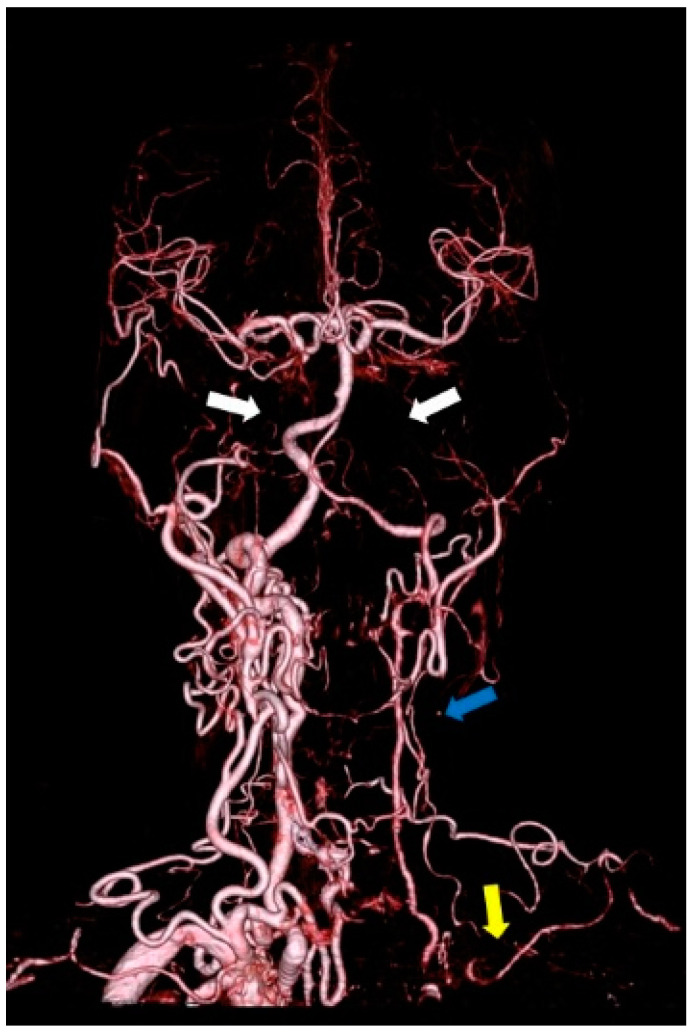
CTA of a 66-year-old woman with TA showing occlusion of the bilateral internal carotid arteries (white arrow), left CCA (blue arrow), and left subclavian artery (yellow arrow).

Figure 1 shows a CTA image of a 66-year-old woman with TA. The image shows occlusion of the bilateral internal carotid arteries, left CCA, and left subclavian artery. A study comparing conventional DSA and CTA in 10 patients with TA revealed that CTA was more useful in identifying the extent of the lesion because it provided information about the vessel wall [27]. In a retrospective study using CTA in 15 patients with clinically diagnosed TA, 11 patients (73%) had major cervical vascular involvement, with the most pronounced changes in the brachiocephalic trunk, left CCA, and left subclavian artery [28]. In a study of 25 patients with suspected TA based on clinical symptoms who underwent CTA and conventional DSA, CTA depicted mural changes, including wall thickening, calcification, and mural thrombi, not seen with conventional angiography. The sensitivity and specificity of CT angiography in the diagnosis of Takayasu arteritis were 95% and 100%, respectively [29].

#### 2.2.2. Monitoring

The wall thickening of the aorta and the relative post-contrast enhancement ratio of the vessel wall are reportedly useful in assessing the activity of TA [30]. In the venous phase, a double ring enhancement pattern is present with clear enhancement of the outer ring and less enhancement of the inner ring [31]. The double ring enhancement pattern is strongly suggestive of TA. The obvious enhancement of the outer ring represents active inflammation of the media and adventitia, and the lesser enhancement of the inner ring represents swelling of the intima [31]. The low attenuation ring represents low attenuation of the intima between the outer wall of the augmented vessel and the opaque blood inside the vessel. The specificity of using this sign to assess disease activity is 100%, and the sensitivity is 34% to 57% [32].

### 2.3. Magnetic Resonance Imaging (MRI)/Magnetic Resonance Angiography (MRA)

#### 2.3.1. Diagnosis

**Figure 2:**
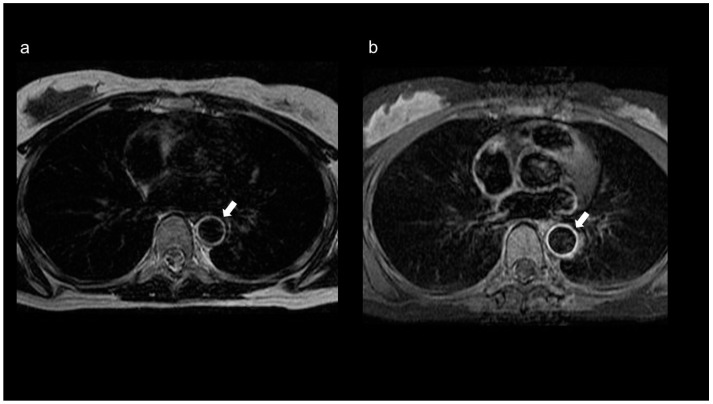
MRI of an 18-year-old female patient with TA. (**a**) The T2-weighted image shows a ring-shaped high signal along the intima in the aortic wall (arrow), and (**b**) the contrast-enhanced T1-weighted image shows a contrast effect in the aortic wall (arrow).

MRA is widely used to detect carotid artery stenosis and reportedly has a higher detection rate than DSA, especially for mild stenosis [33]. The European League Against Rheumatism recommends MRI as the first imaging test for patients with suspected TA because it can evaluate both inflammation of the vessel wall and changes in the vessel lumen. Additionally, because TA is more common in younger patients, tests with less radiation exposure are preferred [34]. MRI of patients with TA shows concentric thickening of the wall, wall mural contrast enhancement on T1-weighted images, and edema on T2-weighted images (Figure 2a,b) [35]. MRA can also evaluate arterial lumen narrowing, occlusion, and aneurysm formation [35,36]. In a study on the diagnostic accuracy of contrast-enhanced three-dimensional MRA for TA when conventional DSA was used as the reference, the sensitivity and specificity of contrast-enhanced three-dimensional MRA were 100% [37].

#### 2.3.2. Monitoring

Choe et al. [38] reported that the disease activity determined using contrast-enhanced MRI was concordant with the clinical findings in 88.5% of patients, with the erythrocyte sedimentation rate in 92.3% of patients, and with the C-reactive protein concentration in 84.6% of patients. An MRI study using gadofosveset trisodium as an intravascular contrast agent showed high sensitivity (100%) and specificity (89%) in differentiating active and inactive TA [39]. Assessment of TA activity by MRI should be done carefully because false-positive results are often seen [40]. The usefulness of edema-enhanced MRI as the sole guide to disease activity and treatment of TA is unclear because of the inconsistencies in the presence or absence of vessel edema and subsequent anatomical changes [41].

### 2.4. Duplex Ultrasonography (DUS)

#### 2.4.1. Diagnosis

**Figure 3:**
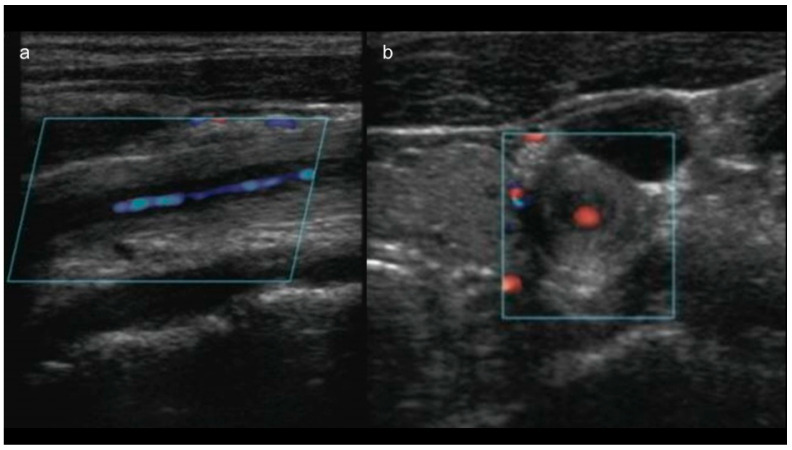
(**a**) Long-axis and (**b**) short-axis view of color Doppler ultrasonography of a 14-year-old female patient with TA showing the “macaroni sign”: circumferential wall thickening of the CCA.

DUS is a reliable tool for characterizing inflammation in the vessel wall and for monitoring hemodynamic changes in response to treatment in patients with TA [42]. Maeda et al. [43] reported that in patients with active TA, B-mode ultrasonography reveals a long segment with homogeneous, mid-echogenic circumferential arterial wall thickening, termed the “macaroni sign” (Figure 3a,b). In one meta-analysis, the pooled sensitivity of ultrasonography for TA was 81%, and the pooled specificity was 100% compared with clinical criteria [32]. In addition, as stated by the authors, this extremely high specificity can be explained by the fact that the studies included in the meta-analysis were case-control designs, comparing patients with longstanding TA versus healthy controls or patients with systemic lupus erythematosus [32]. Therefore, the specificity of DUS for TA is likely overestimated [32].

#### 2.4.2. Monitoring

Svensson et al. [42] reported that patients with clinically active TA had a markedly increased intima-media thickness, increased vessel diameter, intramural arteries, and hypo-echogenic areas interpreted as edema of the vessel wall. They proposed that the Takayasu ultrasound index (which is a summary of the maximum intima-media thickness measurements of the left and right CCAs, brachiocephalic trunk, and aortic arch divided by the number of vessels with reliable intima-media thickness measurements) may be useful for detecting the activity of TA [42]. 

### 2.5. Positron Emission Tomography (PET)

#### 2.5.1. Diagnosis

**Figure 4:**
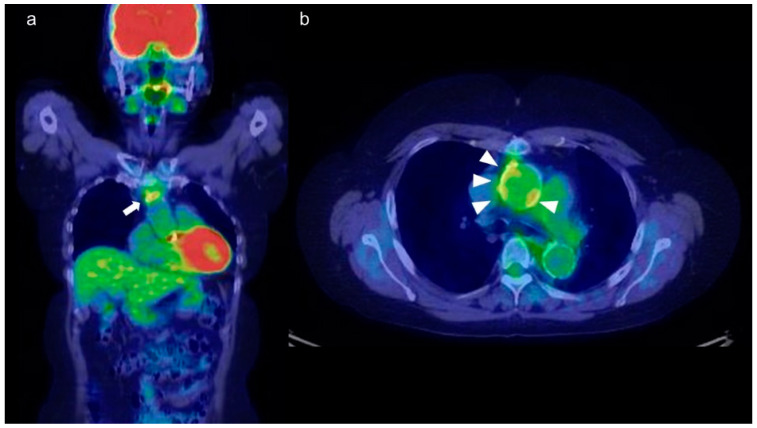
(**a**) Coronal and (**b**) axial view of FDG-PET-CT of a 55-year-old woman with TA. FDG is abnormally accumulated in the vessel wall of the ascending aorta (a: arrow, b: arrowhead).

18F-Fluorodeoxyglucose (FDG)-PET has been used for the diagnosis and assessment of TA (Figure 4a,b) [44]. FDG-PET is often used in conjunction with CT. The transport of FDG in capillaries correlates with glucose uptake. Because activated white blood cells have increased glucose metabolism, FDG-PET is frequently used in infectious and noninfectious inflammatory diseases [44]. It provides functional information on the metabolic activity of organs and tissues. Therefore, FDG-PET can be used before morphological abnormalities and inflammatory edema develop, contributing to early diagnosis [45]. FDG-PET is also reportedly useful for diagnosis and evaluation of disease activity in patients who have TA with atypical clinical manifestations [46]. Several cases of TA diagnosed by FDG-PET in patients with fever of unknown origin have been reported [47,48].

#### 2.5.2. Monitoring

FDG-PET is very useful for early diagnosis and evaluation of disease activity in patients with TA [32]. In one meta-analysis, the pooled sensitivity and specificity of FDG-PET for disease activity compared with clinical assessment were 81% (95% CI: 69–89%) and 74% (95% CI: 55–86%), respectively [32]. In two studies, follow-up FDG-PET data showed an improvement in FDG-PET abnormalities after treatment, and the improvement in FDG-PET findings was consistent with clinical activity [49,50]. Tezuka et al. [51] defined the maximum standardized uptake value (SUV_max_) as the maximum uptake value in the manually drawn volume in the vascular uptake region and reported that SUV_max_ was significantly higher in patients with active TA than inactive TA and without vasculitis, with a sensitivity of 92.6% and specificity of 91.7% for evaluating active TA. In a study of FDG-PET in patients scheduled for carotid endarterectomy (CEA), FDG uptake in carotid plaques was measured as the ratio of the plaque to blood concentration (target/background ratio) [52]. A strong correlation was found between the mean FDG uptake (mean target/background ratio) and the mean degree of inflammation (mean CD68 staining rate) in carotid pathology specimens [52]. The European Association of Nuclear Medicine recommends use of the target/background ratio instead of the SUV for quantification of FDG uptake in atherosclerotic plaques [53]. This is because use of the ratio of the two measurements reduces the effect of patient weight, radioactive dose, and acquisition time errors on quantification of the signal [53]. The PET vascular activity score is a new PET-based parameter created by integrating visual scores of nine susceptible major arteries and can quantitatively reflect the global inflammatory burden [54]. This score is reportedly superior to the SUV_max_ in qualitative and quantitative assessment of TA activity [55].

## 3. Comparative Analysis of Available Imaging Techniques

The main advantages and disadvantages of conventional imaging techniques are summarized in Table 1. Because DSA is highly invasive, it is being used less frequently for diagnosis [26]. Although CTA is useful for diagnosis [27,28,29], it uses radiation and is therefore not optimal for regular monitoring. MRA is considered useful as the first test for diagnosis because it is noninvasive and does not involve exposure to radiation [34]; however, its usefulness for monitoring has not yet been established [40,41]. DUS can clearly show the CCA, vertebral and subclavian arteries, brachiocephalic trunk, renal arteries, and other frequently involved vessels. However, the descending thoracic aorta can only be depicted by transesophageal ultrasonography [56]. DUS is considered useful for both diagnosis and monitoring because it is highly accessible, inexpensive, noninvasive, and radiation-free; notably, however, it is more subjective than other tests [42]. FDG-PET may be particularly useful as a monitoring method [32,44], but it has low accessibility and involves radiation exposure. 

## 4. Novel Imaging Techniques for TA

### 4.1. Optical Coherence Tomography (OCT)

OCT is used to obtain tomographic images based on the coherence of light. It uses infrared light, which confers good resolution but has less-than-optimal tissue penetration [57,58]. OCT can be used to clarify ambiguous ultra-sonographic and angiographic images; by providing detailed microstructural information on plaques, OCT can identify the features of vulnerable carotid plaques and possible defects after stent implantation. It can also be used to assist carotid artery stenting [59,60,61]. In one study, OCT detected 97% of lesions in patients with intracranial atherosclerotic stenosis [62], indicating high sensitivity. OCT can assess the severity of stenosis and guide treatment, has good spatial resolution, and can determine vessel size and plaque morphology [63]. Several case reports have documented the usefulness of OCT for coronary artery evaluation in patients with TA [64,65]. The limitations of OCT are its invasive nature and use as an intravascular imaging method because it only penetrates to a depth of about 2 to 3 mm.

### 4.2. Infrared Thermography

Because of a local acute inflammatory response, atherosclerotic plaques cause a localized temperature increase [66]. A histological study of 48 patients who underwent CEA showed an inverse correlation between the thickness of the fibrous cap of the plaque and the surface temperature of the plaque [66]. If the carotid artery is partially or completely occluded, effective blood perfusion to the skin tissues of the face and forehead will be adversely affected in contrast to normal carotid artery blood flow, and the skin temperature in the area will also be affected. In a study using infrared thermography, 57% of 30 patients with angio-graphically proven stenosis showed unilateral forehead cooling of ≥0.7 °C [67]. Two provocative tests, the facial cooling and head clamp tests, increased the sensitivity to 83% [67]. In another study, ocular temperature was negatively correlated with the degree of carotid stenosis in 24 patients (r = −0.67, *p* < 0.001) [68].

One case report described a 39-year-old patient with TA who showed no abnormalities on contrast-enhanced CT and carotid DUS but exhibited increased thermal retention in the left carotid artery and aortic arch on infrared thermography [69]. The symptoms improved after treatment with steroids, and the abnormalities disappeared on repeat infrared thermography performed 6 months after the treatment [69]. Thus, infrared thermography may also be useful for monitoring TA. Limitations of thermal imaging cameras include the fact that thermal imaging cameras with high spatial resolution are expensive, interpretation requires extensive training, and the working environment (e.g., temperature, humidity, and airflow) can significantly affect the results.

### 4.3. Contrast-Enhanced Ultrasonography (CEUS)

**Figure 5:**
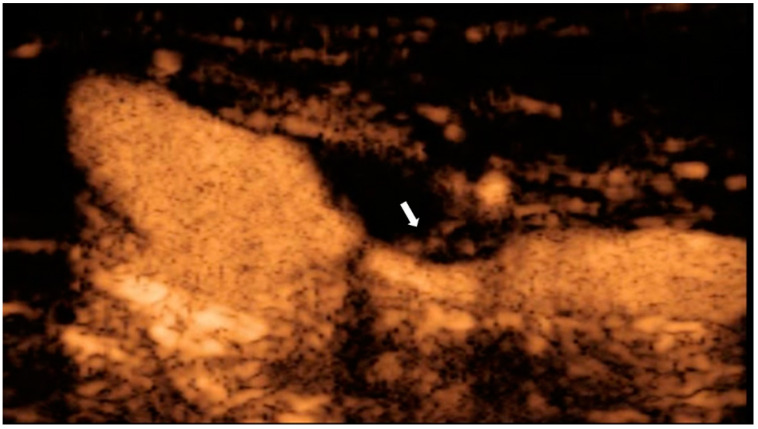
CEUS of a 71-year-old man with asymptomatic severe carotid stenosis. Contrast inflow into the plaque is observed (arrow), suggesting the presence of neovascularization.

Intraplaque hemorrhage has been reported to be associated with histologically disrupted plaque surfaces [70]. Histological neovascularization predicts vulnerability of carotid plaques [71,72,73], and because neo-vessels are immature and fragile, local inflammatory injury or shear stress from the arterial lumen causes them to collapse, leading to intraplaque hemorrhage [72,74]. CEUS provides real-time images of microbubbles, which serve as intravascular tracers that enter the plaque from the lumen and adventitia through neo-vessels (Figure 5) [75,76,77]. Recent studies have shown that visual or quantitative evaluation of contrast effects using CEUS can be used to assess the histopathology of carotid plaque neovascularization, suggesting that high contrast effects in plaque may reliably predict the presence of abundant neovascularization, plaque rupture, and intraplaque hemorrhage [72,74,75,78,79]. We reported that preoperative CEUS predicted micro-embolic signals on transcranial Doppler during carotid artery exposure in 70 patients who were candidates for CEA with a sensitivity of 90% and specificity of 76% [80]. We also reported that the signal intensity of plaques on MRI was associated with the contrast effect on CEUS in patients with severe carotid artery stenosis (≥70%) [81]. In a retrospective study of 71 patients with TA undergoing carotid CEUS, a significant correlation was found between the CEUS results and clinical disease activity [82]. In addition, in 22 patients who underwent both CEUS and FDG-PET, the CEUS results were correlated with vascular FDG uptake [82]. When vascular inflammation was defined as FDG uptake of visual grade ≥ 2, carotid CEUS showed 100% sensitivity and 80% specificity [82]. According to the receiver operating characteristic analysis, the combination of CEUS parameters and the erythrocyte sedimentation rate was able to distinguish between active and inactive TA with a sensitivity of 81.1% and specificity of 81.5% [83]. Limitations of CEUS include loss of the advantages of ultrasound (such as noninvasiveness and ease of use) because of the use of an ultrasound contrast agent as well as the need for training to perform the test and interpret the results [84].

### 4.4. Superb Microvascular Imaging (SMI)

**Figure 6:**
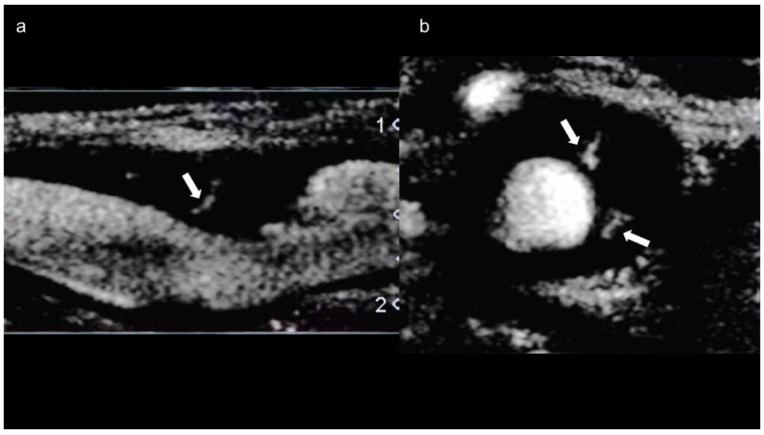
SMI of a 64-year-old man with asymptomatic carotid stenosis. Blood flow signals are seen in both (**a**) long-axis (arrow) and (**b**) short-axis (arrows) images.

SMI is a novel vascular imaging mode that allows for visualization of low-velocity microvascular flow [85]. The SMI technique for motion artifact-specific characteristic analysis enables filtering that eliminates only the motion artifact and facilitates visualization of low velocity blood flow [84]. One study showed that moderate to severe intraplaque neovascularization detected by SMI was more prevalent in subjects with a history of stroke or TIA or with thicker plaques [86]. SMI is a promising noninvasive alternative to CEUS for the assessment of carotid plaque stability [87] and may help prevent ischemic stroke (Figure 6a,b) [88]. We reported that preoperative SMI for cervical carotid artery stenosis predicts the development of micro-embolic signals on transcranial Doppler during exposure of the carotid arteries in CEA [89]. SMI is a simple, safe, and noninvasive technology that has excellent agreement with CEUS [90] but does not require a contrast agent [84]. In two recent case reports of, SMI facilitated detection of neovascularization of the arterial wall without contrast agent in patients with TA [91,92]. Carotid artery neovascularization detected by SMI may be a marker of disease activity in patients with TA [93]. Limitations of SMI are the current lack of clinical standards and the need for training to perform the test and interpret the results [94].

## 5. Conclusions and Perspectives

Early detection and monitoring of TA with appropriate imaging techniques are very important to prevent complications such as stroke and to improve the prognosis. Although conventional imaging techniques are reportedly useful in diagnosis and/or monitoring, all of them have specific advantages and disadvantages; therefore, the technique should be chosen based on the purpose of the test (diagnosis or monitoring), availability, and the patient’s characteristics. MRA and DUS are useful to avoid radiation exposure, especially in younger patients. However, although MRA is recommended for diagnosis, its usefulness as the sole guide for disease activity and treatment of TA is unclear because of inconsistencies in the presence of angioedema and subsequent anatomical changes. Conversely, DUS is highly dependent on the examiner’s skill level, is more subjective than other tests, cannot depict vessels with calcification, and cannot be used to observe vessels throughout the body. Novel imaging techniques such as OCT, infrared thermography, CEUS, and SMI may be useful to improve the accuracy of diagnosis and/or monitoring of TA. Although the available data are promising, only small studies have been performed to date, and future studies involving larger patient series or cohorts are needed. Moreover, other novel imaging techniques, such as photoacoustic tomography, should be tested in the diagnosis or monitoring of TA [95]. Other approaches, such as nuclear magnetic resonance spectroscopy-based serum metabolomics study, may also find a place in monitoring of disease activity in the future, but the evidence is limited at present [96].

## Figures and Tables

**Table 1:** Comparative analysis of conventional imaging techniques in diagnosis and monitoring of TA.

Modality	Accessibility	Ease of Use	Purpose/Use	Radiation	Principal Limitations
*DSA*	Low	Low	Diagnosis/Treatment	Yes	Invasive; lack of information on the vessel wall
*CTA*	High	High	Diagnosis/Monitoring	Yes	Cannot be used in patients with renal failure or allergies to contrast media
*MRI/* *MRA*	Low	High	Diagnosis	No	Cannot be performed when some types of metals are present in the body or in patients with claustrophobia
*DUS*	High	High	Diagnosis/Monitoring	No	Examiners’ technical proficiency strongly affects the result; subjective; acoustic shadow
*PET*	Low	High	Diagnosis/Monitoring	Yes	Lack of criteria for positivity (FDG uptake); low resolution for small vessels

## Data Availability

Not applicable.
